# Karst study of Jinfo Mountain based on image analysis

**DOI:** 10.1016/j.heliyon.2023.e19657

**Published:** 2023-08-30

**Authors:** Honghai Kuang, Jinghao Li, Xiyao Wang

**Affiliations:** School of Geographical Sciences, SouthWest University, Tiansheng Road 2, Beibei, Chongqing, China

**Keywords:** Karst, Carbonate, Image analysis method

## Abstract

The KDR (karst development rate) of rocks and their PCR(porosity of carbonate rocks) are common research topics in Jinfo Mountain. The use of traditional carbonate research methods (TCRMs) for karst studies has been shown to be costly and time-consuming. Therefore, this study attempted to find a new, reliable, low-cost, and time-saving method for karst research. The Jinfo Mountain area is a typical carbonate rock area that is suitable for karst research. In this study, many images of rock samples from the Jinfo Mountain were obtained using rock-polarizing microscopes, which provided a good basis for the karst study of Jinfo Mountain. Furthermore, in this study, image analysis technology was used to find the karst development rate of rocks and their porosity. To ensure the accuracy of these research results, we compared the research results obtained using the image analysis techniques with those obtained using TCRM. The comparison showed that the image analysis technology is a feasible research techniques for studying karst in the Jinfo Mountain area. Furthermore, it has good reference significance for other karst study outside the Jinfo Mountain area.

## 1 Introduction

Research precedent has been set for karst research in carbonate rock regions. Advances in computer technology have provided new methods for conducting karst research in carbonate rock areas. Many of these karst studies have been conducted using theT (traditional)C (carbonate) R (research) M(method) (TCRM). The popularization of computer technology, however, should not be ignored in continued karst research in carbonate rock areas. As an important feature of computer technology, imaging technology has shown wide application prospects in karst research in carbonate rock areas.

TCRM is a common karst research method [1,2], which has many examples in the study of rock properties and karstification of carbonate rocks [[3], [4], [5]]. The study of carbonate rocks' porosity is essential in the study of karstification in carbonate rocks [[6], [7], [8]] and has many applications [9,10]. Many methods can be used to study carbonate rocks' porosity [[11], [12], [13]]. Some of these studies have illustrated that nontraditional karst research techniques can also be used [4,14]. Furthermore, some studies have used non-TCRMs to study carbonate rock porosity [15,16]. Some recent research advances in other disciplines have been applied to the study of carbonate rock porosity [[17], [18], [19], [20]].

Scholars have used multi-technical methods for carbonate rock research [21,22]. Some scholars have studied carbonate rock based on mathematical modeling [[23], [24], [25]]. Several algorithms have been applied in the study of carbonate rock areas [[26], [27], [28], [29]]. Other scholars also have tried to apply image analysis techniques in the study of carbonate rocks [[30], [31], [32]]. Some studies have integrated image analysis technology with three-dimensional (3D) modeling technology to study carbonate rocks [[33], [34], [35], [36], [37]]. Other studies have shown that image binarization is promising in the study of carbonate rock images [[38], [39], [40], [41], [42]]. Additionally, research results have shown the importance of comparing the results of multiple research methods with those of TCRM [[43], [44], [45], [46], [47]]. Recent studies have shown that it is essential to use image techniques in rock research [[48], [49], [50], [51], [52]].

Whether the image analysis **techniques** can be used to study the karst development rate in carbonate areas should be verified by repeated studies. The research method of image analysis conducted in the Jinping area also should be verified in other carbonate rock areas. Currently, few precedents have been set to the karst study using the image **techniques**. Therefore, it is impossible to confirm whether the research used in the Jinping area can be applied directly to the Jinfo Mountain. The Jinfo Mountain is an ideal carbonate research area. Therefore, in this study, we attempted to verify the research methods used in the Jinping area by applying those same methods to the Jinfo Mountain area.

## 2 Materials and methods

### 2.1 Study site and samples

**Fig. 1:**
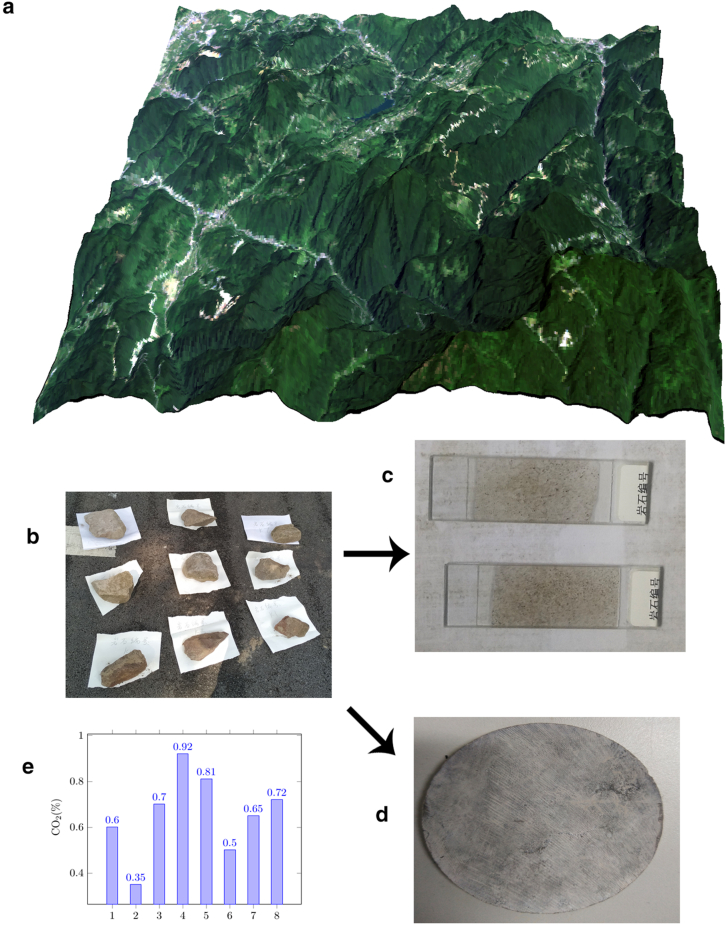
**Jinfo Mountain area and carbonate samples:** (a) 3D model of Jinfo Mountain created using ArcGIS. (b) Carbonate rock samples from the Jinfo Mountain area. (c) Carbonate polarized light microscope slides of Jinfo Mountain carbonate rock samples. (d) A specimen used for the TCRM made from a Jinfo Mountain carbonate rock sample. (e) The carbon dioxide (CO_2_) content of the water samples in the Jinfo Mountain.

The Jinfo Mountain area (Fig. 1(a)) is a suitable karst study area. It has a wide distribution of Karst landform, and karst water is also widely distributed in this area. The main conditions for the occurrence of karstification are distributed throughout the Jinfo Mountain area. From 2001 to the present, Southwest University has conducted a relatively long period of karst research in this area. During this period, a large amount of historical observation data was accumulated. In the karst research conducted by Southwest University in the Jinping area in western Sichuan, the karst research based on image analysis methods obtained relatively good research results. Therefore, in this study, we used the historical observation data for the Jinfo Mountain area to verify the Karst research results obtained using the same carbonate rock image techniques.

### 2.2 Karst study of e Jinfo Mountain using TCRM

Karst research has been conducted in the Jinfo Mountain area for a long time. A significant amount of karst research data has been accumulated for the Jinfo Mountain area. Among the karst research data, the KDR and PCR are the most common types of data. In the Jinfo Mountain area, PCR has been obtained using the gasoline methods. Because the use of gasoline in the laboratory must be authorized by the government, water is generally used instead of gasoline in the laboratory. Most of the carbonate porosity data for the Jinfo Mountain area was obtained through water immersion. Our research process was similar to that conducted using gasoline. The carbonate specimens soaked in water were dried, however, instead of burned. The KDR of the rocks in the Jinfo Mountain was determined primarily by putting the carbonate rock specimen shown in Fig. 1(d) into the karst water near the carbonate rock collection site. The carbonate rock specimens were weighed before being put into the karst water. Then, they were removed from the water, allowed to dry for a few days, and weighed. We calculated the KDR of the rocks using the difference between the initial and final weights and the number of days of immersion.

### 2.3 Karst study of Jinfo Mountain using image analysis

In a karst study in the Jinping area, we found that the PCR in different periods obtained through image analysis had a linear multiplication relationship. This linear multiplication relationship also existed for the KDR of the rocks. The Jinfo Mountain area is similar to the Jinping area. Both the Jinfo Mountain area and the Jinping area have extensive distributions of carbonate rocks (Fig. 1(b)), and karst studies have been conducted for a long time in both areas. Therefore, the image analysis karst research techniques used in the Jinping area should also be applied to the Jinfo Mountain area (Fig. 3(b)). Images of rocks could be obtained from the carbonate slides made for the lithologic analysis collected from the Jinfo Mountain area.

### 2.4 Fitting and approximation of image processing algorithms for images of carbonate rocks

Porosity research on carbonate rock using image analysis has been conducted primarily to obtain the new value of each image pixel point of a rock using an image analysis algorithm. The new value of each image pixel constituted a new matrix. After finding a suitable threshold, the image of the rock was divided into black-and-white binary images using the threshold. This threshold was obtained using statistical methods. From the experience in Jinping, taking the PCR obtained using the TCRM as the target value, we compared the PCR obtained using this threshold value. This is a good research method and gradually should be able to approach the TCRM porosity using the porosity obtained with the threshold value through a target approximation.

## 3 Results

### 3.1 Study of carbonate rocks from Jinfo Mountain using image analysis

In this study, we used the image of a slide of Jinfo Mountain rock (Fig. 1(c)) to conduct karst research according to the image analysis techniques To verify whether the image analysis techniques could be used for karst research of Jinfo Mountain, we used Table 1 to arrange the data given in Table 2, Table 3 for verification.

**Table 1:** Sample data collected at the same location on Jinfo Mountain, 2006–2010. A: Sample number; B: PCR obtained via image analysis of 2006 carbonate slides (%); C: PCR obtained via image analysis of 2010 carbonate slides (%); D: PCR obtained via image analysis of 2010 carbonate slides/PCR obtained via image analysis of 2006 carbonate slides (C/B); E: measured KDR in 2006 (mm/ka); F: measured KDR in 2010 (mm/ka); G: measured KDR in 2010/measured KDR in 2006 (F/E); H: (measured KDR in 2010/measured KDR in 2006)/(PCR obtained via image analysis of 2010 carbonate slides/PCR obtained via image analysis of 2006 carbonate slides) (G/D); and I: (1 − (measured KDR in 2010/measured KDR in 2006)/(PCR obtained via image analysis of 2010 carbonate slides/PCR obtained via image analysis of 2006 carbonate slides)) <20%, 1 − (G/D) <20%.

A	B	C	D	E	F	G	H	I
1–1	0.267	0.357	1.337	21.34	25.72	1.205	0.901	TRUE
1–2	0.223	0.296	1.327	27.56	31.63	1.147	0.864	TRUE
2–1	0.703	0.787	1.119	31.11	27.82	0.894	0.798	FALSE
2–2	0.667	0.697	1.044	25.42	29.33	1.153	1.104	TRUE
2–3	0.553	0.661	1.195	33.71	37.42	1.11	0.928	TRUE
3–1	0.301	0.392	1.302	17.32	22.91	1.322	1.015	TRUE
3–2	0.277	0.403	1.454	15.22	23.42	1.538	1.057	TRUE
4–1	0.355	0.387	1.09	20.89	29.63	1.418	1.3	FALSE
4–2	0.425	0.503	1.183	23.91	35.76	1.495	1.263	FALSE
4–3	0.431	0.523	1.213	25.74	21.42	0.832	0.685	FALSE
5–1	0.692	0.722	1.043	32.58	41.26	1.266	1.213	FALSE
5–2	0.717	0.798	1.112	29.34	31.33	1.067	0.959	TRUE
6–1	0.232	0.295	1.271	25.22	28.98	1.149	0.904	TRUE
6–2	0.331	0.397	1.199	28.16	25.32	0.899	0.749	FALSE
6–3	0.297	0.311	1.047	22.53	28.71	1.274	1.216	FALSE
7–1	0.764	0.882	1.154	30.46	36.22	1.189	1.03	TRUE
7–2	0.651	0.708	1.087	28.72	31.53	1.097	1.009	TRUE
8–1	0.561	0.631	1.124	32.13	37.66	1.172	1.042	TRUE
8–2	0.832	0.872	1.048	29.68	35.23	1.186	1.131	TRUE

**Table 2:** Observation data for carbonates from Jinfo Mountain, 2006–2012.

A	B	C	D	E	F	G	H
1–1	21.34	25.72	1.205	29.37	1.142	−5.2	TRUE
1–2	27.56	31.63	1.147	35.63	1.126	−1.83	TRUE
2–1	31.11	27.82	0.894	39.53	1.421	58.9	FALSE
2–2	25.42	29.33	1.153	31.22	1.064	−7.71	TRUE
2–3	33.71	37.42	1.11	45.66	1.22	9.9	TRUE
3–1	17.32	22.91	1.322	–	–	–	–
3–2	15.22	23.42	1.538	22.91	1.096	−28.7	FALSE
4–1	20.89	29.63	1.418	27.69	0.93	−34.4	FALSE
4–2	23.91	35.76	1.495	29.57	0.826	−44.7	FALSE
4–3	25.74	21.42	0.832	–	–	–	–
5–1	32.58	41.26	1.266	31.24	0.757	−40.2	FALSE
5–2	29.34	31.33	1.0678	33.46	1.0679	0.009	TRUE
6–1	25.22	28.98	1.149	–	–	–	–
6–2	28.16	25.32	0.899	31.96	1.262	40.3	FALSE
6–3	22.53	28.71	1.274	–	–	–	–
7–1	30.46	36.22	1.189	–	–	–	–
7–2	28.72	31.53	1.097	–	–	–	–
8–1	32.13	37.66	1.172	32.95	0.874	−25.4	FALSE
8–2	29.68	35.23	1.186	–	–	–	–

A: Sample number; B: measured KDR in 2006 (mm/ka); C: measured KDR in 2010 (mm/ka); D: measured KDR in 2010/measured KDR in 2006 (C/B); E: measured KDR in 2012 (mm/ka); F: measured KDR in 2012/measured KDR in 2010(E/C); G: (F − D)/D(%); and H: |G |<10%.

**Table 3:** KDR of the rocks in the Jinfo Mountain obtained via image analysis. A: Sample number; B: measured KDR in 2006 (mm/ka); C: measured KDR in 2010 (mm/ka); D: measured KDR in 2010/measured KDR in 2006 (C/B); E: expected KDR obtained using image analysis in 2012 (mm/ka); F: measured KDR in 2012 (mm/ka); G: ((F-E)/E)(%); and H:|G|< 15%. Samples 5-1 and others are false in Table 1, column I. Therefore, we found that the image analysis method was not suitable for these samples.

A	B	C	D	E	F	G	H
1–1	21.34	25.72	1.205	36.43	29.37	−19.37	FALSE
1–2	27.56	31.63	1.147	43.59	35.63	−18.26	FALSE
2–1	31.11	27.82	0.894	36.8	39.53	7.4	TRUE
2–2	25.42	29.33	1.153	26.84	31.22	16.31	FALSE
2–3	33.71	37.42	1.11	41.56	45.66	9.8	TRUE
3–1	17.32	22.91	1.322	–	–	–	–
3–2	15.22	23.42	1.538	18.23	22.91	25.67	FALSE
4–1	20.89	29.63	1.418	24.37	27.69	13.62	TRUE
4–2	23.91	35.76	1.495	32.11	29.57	−7.9	TRUE
4–3	25.74	21.42	0.832	–	–	–	–
5–1	32.58	41.26	1.266	38.15	31.24	−18.11	FALSE
5–2	29.34	31.33	1.067	28.01	33.46	19.45	FALSE
6–1	25.22	28.98	1.149	–	–	–	–
6–2	28.16	25.32	0.899	34.77	31.96	−8.08	TRUE
6–3	22.53	28.71	1.274	–	–	–	–
7–1	30.46	36.22	1.189	–	–	–	–
7–2	28.72	31.53	1.097	–	–	–	–
8–1	32.13	37.66	1.172	36.75	32.95	−10.34	TRUE
8–2	29.68	35.23	1.186	–	–	–	–

**Fig. 2:**
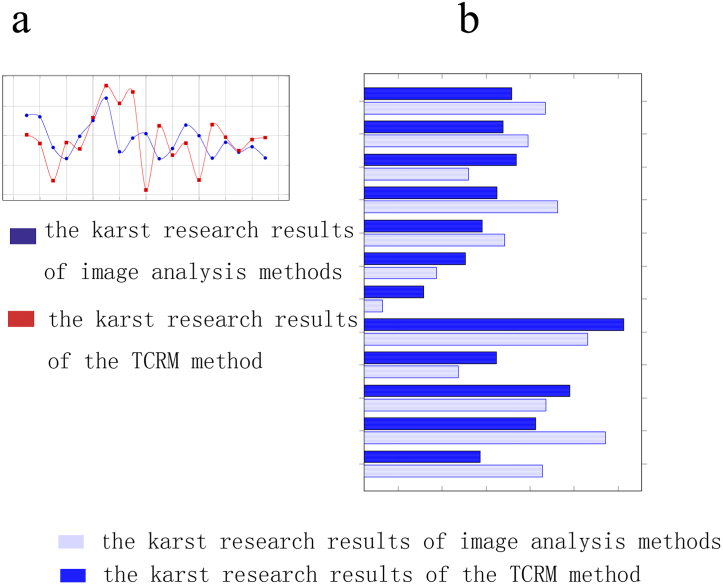
**Comparison of the research results of the image analysis techniques and the TCRM:** (a) Line graph comparing the research results of the image analysis techniques and the TCRM. (b) Histogram comparing the research results of the image analysis techniques and the research results of TCRM.

If the image analysis method is applicable to karst research of Jinfo Mountain, then the linear multiplication value of the PCR obtained using the image analysis techniques and the TCRM should not be drastically different (Fig. 2(a)). The values in Table 1, columns D and G, also should not be very different. From the values in Table 1, columns D and G, the linear multiplication relationship between the image analysis techniques and the TCRM for most of the samples was relatively close. This result indicated that the image analysis techniques can be applied in the Jinfo Mountain area (Fig. 2(b)). Karst studies in other carbonate regions using image analysis techniques also should pass a verification test similar to that outlined in Table 1. After passing this test, we calculated the KDR in 2010 using Table 1, column D, and the carbonate KDR measured in 2006. We found little difference between the calculated results and the carbonate KDR measured in 2010. As shown in Fig. 2, the research results of the image analysis techniques and the TCRM were relatively close.

**Fig. 3:**
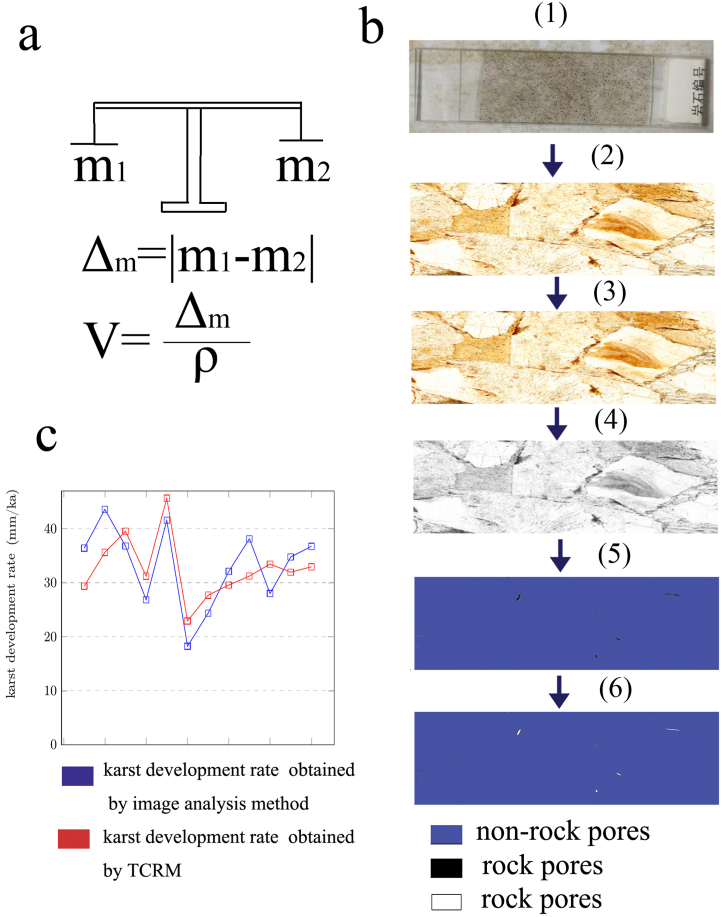
**Karst development rates obtained using the two research methods:** (a) KDR using TCRM. b (1) A carbonate rock slide; b (2) the original image of a rock slide; and b (3) the polarized image preprocessed using Photoshop. b (4) The polarized image in grayscale. b (5) A polarized image converted to a black-and-white image, in which white has been replaced by blue. b (6) The carbonate pore map obtained from the black and white image using imagej2x, in which the white has been replaced with blue. (c) Comparison of karst development rates using the two research methods: the blue line is the KDR obtained using the image analysis techniques, and the red line is the KDR obtained using the TCRM.

### 3.2 Results for the carbonate rocks from Jinfo Mountain using the TCRM

Table 2 compares these experimental data with the results obtained using the image analysis techniques and the observation data for the.rocks of Jinfo Mountain.

Samples 5-1 and others are false in Table 1, column I. Therefore, the image analysis method was not suitable for these samples. As a result, we did not use these samples to perform the calculations reported in Table 2. If Table 2, column H, also was false, no linear multiplication relationship existed between the karst development rates at the locations where the carbonate samples were collected, and the local karst development rates had changed. Therefore, if Table 2, column H, was false, the image analysis techniques could not be used for karst research.

### 3.3 Results for the KDR of the rocks from Jinfo Mountain using image analysis

We conducted carbonate research using image analysis mainly to obtain the PCR. We obtained the karst development rate by analyzing the linear multiplication relationship of the PCR at the same location. Therefore, the karst development rate could be obtained only for the samples tested in Table 1, Table 2 using the image analysis techniques. According to Table 1, Table 2, we obtained the KDR of the carbonate rocks in the Jinfo Mountain given in Table 3 using image analysis.

## 4 Discussion

### 4.1 Comparison of the image analysis method and the TCRM

The application of image analysis technology to karst research has been limited by its unreliable results. Therefore, it is necessary to find a correct method to verify the reliability of its results (Fig. 3(a)). Furthermore, it is generally believed that the research results of TCRMs are reliable. Therefore, the TCRM is an excellent method to verify the results of image. The karst studies obtained using the image analysis techniques should not be very different from those obtained using the TCRM. If the results obtained using the image analysis techniques are significantly different from the results obtained using the TCRM, then the algorithm of the image analysis techniques needs to be improved. In this study, the karst research data obtained using the image analysis techniques and the TCRM were composed (Table 3). As can be seen from Tables 3 and if column H is used as the criterion, 6 of the 19 samples can be considered to be correct, and the accuracy rate is 31.57%. This accuracy rate exceeds the accuracy rate of human naked eye judgment. If the number of samples used in the analysis method in the Jinfo Mountain area increases, the accuracy of the study should also improve. As shown in Fig. 3(c), the karst development rates are relatively close.

### 4.2 Methods to improve the accuracy of karst research results using carbonate rock image analysis

**Fig. 4:**
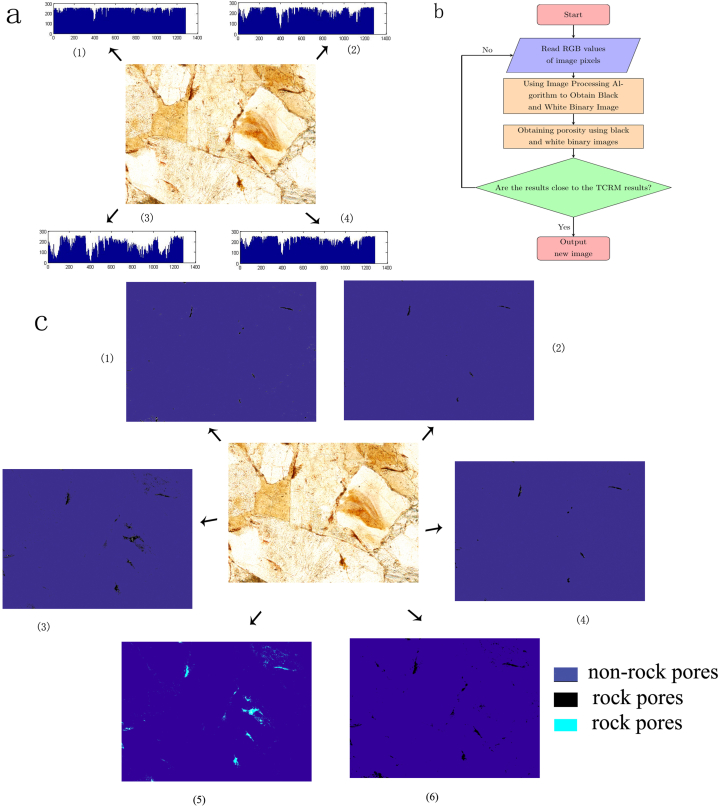
**Karst study of Jinfo Mountain using image analysis:** a (1) The R-value curve of the image of a rock from Jinfo Mountain. a (2) The G-value curve of the image of a rock from Jinfo Mountain. a (3) The B-value curve of the image of the Jinfo Mountain rock. a (4) The gray-value curve of the image of a rock from Jinfo Mountain. (b) A flowchart of the image analysis of rocks from Jinfo Mountain for karst research. c (1) A binary map of the pores obtained using the R-value, in which the white areas have been changed to blue. c (2) A binary map of the pores obtained using the G-value, in which the white areas have been changed to blue. c (3) A binary map of the pores obtained using the G-value, in which the white areas have been changed to blue. c (4) A binary map of the pores obtained using the gray-value, in which the white areas have been changed to blue. c (5) The carbonate pore binary map obtained using the Jinping finite automaton [40], in which the white areas have been changed to blue to distinguish them from the background color. c (6) A binary map of the carbonate rock pores obtained using the Jinping finite automaton [40], in which the white areas have been changed to blue to distinguish them from the background color.

The results obtained from previous karst image analysis studies were not necessarily accurate. In karst image analysis studies, it is necessary to improve the algorithm step by step to improve the accuracy of the research results (Fig. 4(b). As shown in Fig. 4(a), many results in karst research have been obtained using the image analysis techniques. The study obtained using the TCRM are generally considered to be accurate. In this study, we used the results obtained with the TCRM as target values. After the image analysis method had determined the algorithm, we modified the settings of the operator. The research results of the image analysis techniques gradually approached those of the TCRM. The process of getting closer to the result was an iterative process of the algorithm (Fig. 4(c)). The iteration of the finite automaton should pay attention to the selection of the iteration curve. The iteration curve should be a curve family with open-source functions available on a code-hosting website, which should significantly improve the efficiency of the algorithm iterations.

## 5 Conclusions

The advantages of using image analysis methods for carbonate karst studies are obvious. Compared with the TCRM, the time-saving advantage of using the image analysis method for carbonate karst research was obvious. Studies that take days to complete using the TCRM can be conducted in just minutes using image analysis. Compared with the TCRM, the cost advantage of using the image analysis method for carbonate karst research was obvious. Because carbonate slides have to be used for rock property analysis in the TCRM, there is no additional cost to use carbonate slides for karst research. Because the processing cost of carbonate rock slides is low, carbonate rock slides can be processed as long as fragments of carbonate rock samples are available. Some karst studies of carbonate rocks conducted using the TCRM are labor-intensive, whereas image analysis methods are significantly less labor-intensive. Compared with karst studies of carbonate rocks performed using the TCRM, the image analysis method allows researchers to quickly repeat a given study using open-source code. Because of the characteristics of open-source code, the image analysis method allows other researchers to participate in the research or assist more quickly than when using the TCRM. Compared with the TCRM, the image analysis techniques can take advantage of open-source code, which other scholars can also access. Compared with the TCRM, the image analysis techniques can benefit from the latest achievements in disciplines, such as artificial intelligence and big data. The image analysis techniques enables more scholars from other disciplines to enter the field of karst research.

The following points should be noted when extending the study to other Karst areas. The purity of the rocks should be relatively high. It is not difficult to collect samples of carbonate rocks. The processing cost of local carbonate slides and specimens is low. Local karst observation research has been conducted for a long time, and therefore enough historical research data can be used for verification. The local karst water and hydrochemical indicators are similar. Carbonate rock areas that meet the noted conditions should also be suitable for use of the image analysis techniques. To extend the study to other karst areas, we must also pay attention to the karst study of the image analysis techniques. To ensure that the application of this research method is meaningful, the accuracy rate of the Karst study of the image analysis techniques should be higher than the accuracy rate of local researchers who make judgments with the naked eye.

## Author contribution statement

Honghai Kuang: Conceived and designed the experiments; Performed the experiments; Analyzed and interpreted the data; Contributed reagents, materials, analysis tools or data; Wrote the paper.

Jinghao Li: Contributed reagents, materials, analysis tools or data.

Xiyao Wang: Contributed reagents, materials, analysis tools or data.

## Data availability statement

Data associated with this study has been deposited at https://doi.org/10.5281/zenodo.6596935.

## Declaration of competing interest

The authors declare that they have no known competing financialinterestsor personal relationships that could have appeared to influence the work reported in this paper.
